# Band Engineering and Majority Carrier Switching in Isostructural Donor–Acceptor Complexes DPTTA‐F*_X_*TCNQ Crystals (*X* = 1, 2, 4)

**DOI:** 10.1002/advs.201902456

**Published:** 2019-11-26

**Authors:** Yingying Liang, Yunke Qin, Jie Chen, Weilong Xing, Ye Zou, Yimeng Sun, Wei Xu, Daoben Zhu

**Affiliations:** ^1^ Beijing National Laboratory for Molecular Sciences Key Laboratory of Organic Solids Institute of Chemistry Chinese Academy of Sciences Beijing 100190 China; ^2^ University of Chinese Academy of Sciences Beijing 100049 China

**Keywords:** band engineering, donor–acceptor complexes, majority carrier switching, narrow gap, thermoelectric material

## Abstract

Three isostructural donor–acceptor complexes DPTTA‐F*_X_*TCNQ (*X* = 1, 2, 4) are investigated experimentally and theoretically. By tuning the number of F atoms in the acceptor molecules, the resulting complexes display a continuous down shift of the valence band maximum, conducting band minimum, and optical bandgap. The majority carriers convert from hole (DPTTA‐F_1_TCNQ), balanced hole, and electron (DPTTA‐F_2_TCNQ) to electron (DPTTA‐F_4_TCNQ). This result shows that band engineering can be realized easily in the donor–acceptor complex systems by tuning the electron affinity of the acceptor. The bandgaps of these three complexes vary from 0.31 to 0.41 eV; this narrow bandgap feature is crucial for achieving high thermoelectric performance and the unintentional doping in DPTTA‐F_4_TCNQ leads to the effective suppression of the bipolar cancelling effect on the Seebeck coefficient and the highest power factor.

## Graphical abstract

Variation of the number of F atoms in the acceptor units of a series of isostructural‐crystal DPTTA‐F*_X_*TCNQ complexes leads to continuous tuning of the energy gaps and the species of majority carriers. Efficient band structural engineering effects induced by tuning the electron affinity of the acceptor component are thus shown.

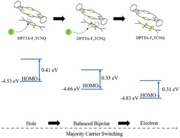

Efficient band engineering (turning of the bandgap, band‐edge energies, and profiles of valance band and conducting band) of organic semiconductors is still a great challenge in organic electronics, although it has already been a general strategy to vary the electronic and optical performance of inorganic semiconductors to meeting the requirement on specialized device applications.^1^ For organic semiconductors, molecular tailoring is the general strategy to tune the energy level of frontier orbitals, structural geometry and so on for adjusting their optical and electronic properties.^2^ Besides, recent advances on donor–acceptor (DA) complexes especially the mixed‐stack crystals revealed that they could provide a feasible approach for achieving ambipolar transport,^3^, ^4^, ^5^, ^6^ strong photoluminescence,^7^ ferroelectricity,^8^ photoresponsibility,^9^ and so on. All these physical properties appeared with the variations of the electronic structures of this binary composed material system, showing the fact that combinations of donor and acceptor molecules with the mixed‐stack fashion provided an alternative avenue for band engineering and tunable physical characters which is more feasible comparing with that of chemical tailoring of single component materials.^10^ Especially for the one acceptor (donor) with few different donors (acceptors) systems.^11^ It should be noted that there are still some goals that seem impossible to achieve for single component organic semiconductors. such as extremely narrow‐gap materials with a bandgap around 10 *K*_b_ *T* (*K*_b_ refers to Boltzmann constant and *T* is the absolute temperature). Such character is critically important for inorganic semiconductor to achieve superior thermoelectric (TE) performance, but still unexplored for organic counterpart.^12^ Currently, there are growing interests in developing small‐molecule and polymer‐based TE materials.^13^ But the major obstacle is their inferior power factors (PF = *σS* ^2^, σ and *S* are electrical conductivity and Seebeck coefficient, respectively), which is due to the formation of gapless polaron band under the scenario of highly doped state.^14^ So, narrow‐gap materials should be investigated for exploring the possibility of enhancing PF by simultaneously achieving high electrical conductivity and Seebeck coefficient just like what observed in the conventional inorganic TE materials.

Recently, we reported two mixed stack DA complexes DPTTA‐F*_X_*TCNQ (DPTTA = meso‐diphenyl tetrathia[22]annulene[2,1,2,1], F*_X_*TCNQ = fluorinated derivatives of 7,7,8,8,‐tetracyanoquinodimethane, *X* = 2 and 4, **Scheme** 1), which displayed stable ambipolar transport properties under ambient condition with electron and hole mobilities exceeding 0.1 cm^2^ V^−1^ s^−1^.^6^ Such performance, especially the simultaneous electron and hole injecting through the gold electrodes implies the narrow bandgap character of the two materials.^15^ Furthermore, the crystal data show that the two complexes possess identical symmetry and cell parameters indicating an isostructural feature, which makes them an ideal model system for systematical investigation on the band structure engineering of DA complexes. Here, a new complex DPTTA‐F_1_TCNQ (F_1_TCNQ = 2‐fluoro‐7,7,8,8‐tetracyanoquinodimethane, Scheme 1) is added in to this system. Besides the amibipolar transport properties showing in the corresponding single crystal field effect transistors (SCFETs), some important parameters related to the electronic structure were investigated experimentally and theoretically, including energy gap derived from optical spectroscopy, active energy of the charge carriers extracted from the conductivity‐temperature relationship, as well as the work function and valance band edge determined by UPS. The bandgaps vary from 0.31 to 0.41 eV with charge carrier concentration ranging from 1.31 × 10^16^ to 8.15 × 10^17^ cm^−3^. The majority carriers convert from hole (DPTTA‐F_1_TCNQ), balanced hole and electron (DPTTA‐F_2_TCNQ) to electron (DPTTA‐F_4_TCNQ). Band structures, as well as the intermolecular electronic coupling were calculated. The observed tuning of the majority carrier species and electronic structures show that the band engineering of the DA complexes can be realized by tuning the electron affinity of the acceptor component.

**Scheme 1:**
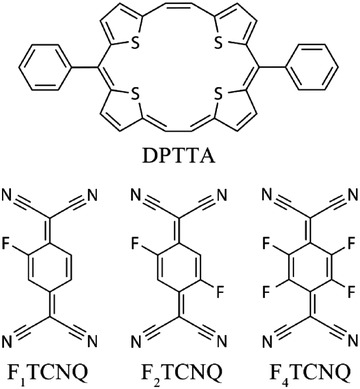
Chemical structure of F*_X_*TCNQ and DPTTA.

Evaluation of the single crystal structure is crucial for understanding the structure–property relationships of organic semiconductors. DPTTA, a sulfur‐bridged annulene, is a typical electron donor and was demonstrated to be one of the most suitable donor components for construction of DA complexes.^4^ TCNQ and its derivatives have been identified as one of the best acceptor molecules for the construction of DA complexes due to their planar configuration, good crystallinity, and tunable energy level.^16^ The bulk single crystals of the DA complexes formed by DPTTA and F*_X_*TCNQ suitable for single crystal structure analysis grew through a diffusion method according to the procedure reported previously.^6^ The crystallographic data are collected in Table S1 in the Supporting Information. The complexes all crystallized in the triclinic unit cell belonging to *P* $\bar{1}$ space group. It should be noted that the F atoms in DPTTA‐F_1_TCNQ disordered over 2, 5‐positions and in DPTTA‐F_2_TCNQ over 2, 3, 5, and 6‐positions of the quinoid ring, respectively. So, F_1_TCNQ presents as a pseudo‐F_2_TCNQ molecule and F_2_TCNQ as a pseudo‐F_4_TCNQ molecule in the complex crystals, which leads to the almost identical structure of these crystals not only in the cell parameters but also the relative position between the donor and the acceptor molecules. **Figure** 1a–c shows the molecular packing patterns of the three DPTTA based complexes. The acceptor molecule stacks over the center of the DPTTA core to maximize the π–π interaction between donor and acceptor. The bond length of b, c, and d in F*_X_*TCNQs are sensitive to the charge transfer (CT) process (see the note of Table S2 in the Supporting Information for the bond labeling). The bond length ratio *r* value (*r* = c/(b + d)) can estimate the degree of charge transfer^17^

(1) $$\mathrm{DCT}=\frac{r_{\mathrm{CT}}-r_{0}}{r_{-1}-r_{0}}$$

where DCT is the degree of charge transfer, the subscripts CT, −1, and 0 refer to charge transfer compound, the anion, and the neutral molecule. The data in Table S2 in the Supporting Information show that in the complexes, the *r* values are in between that of the neutral and fully ionized acceptor molecules, and the CT degrees derived to be 0.111, 0.213, 0.923 for DPTTA‐F_1_TCNQ, DPTTA‐F_2_TCNQ, and DPTTA‐F_4_TCNQ, respectively. The CT ratio increases with the number of F atoms in acceptor molecules, which can be further confirmed by the IR spectra (Figure S1, Supporting Information). It should be noted that this result is different from what we previewed in DPTTA‐TCNQ complex, where the molecules maintain in the neutral state.^4^ Figure 1d–f show the distance between the donor and the acceptor along the mixed‐stack direction, which are 3.46, 3.49, and 3.51 Å (half of the interplane distance of adjacent annulene rings) for DPTTA‐F_1_TCNQ, DPTTA‐F_2_TCNQ, and DPTTA‐F_4_TCNQ respectively, indicating the existence of intermolecular π–π interaction in the DA pairs.^18^ The slight increase of interplanar distance is due to the distortion degree variation of the coplanar conformation of tetrathia[22]annulene[2,1,2,1] center core caused by the ionization. CT in DA complexes acts as a strong driving force to promote intermolecular orbital overlap and to tune the energy levels of the resulting complexes simultaneously.^19^

**Figure 1:**
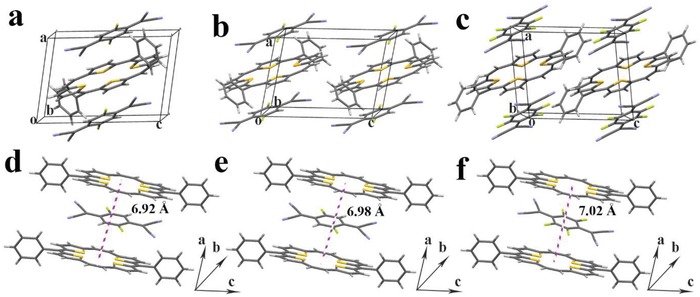
a–f) Molecular stacking and π‐spacing of: a,d) DPTTA‐F_1_TCNQ, b,e) DPTTA‐F_2_TCNQ, and c,f) DPTTA‐F_4_TCNQ.

Charge carrier mobility, electrical conductivity, and Seebeck coefficient are the most important parameters reflecting the semiconducting behaviors of the CT complexes. Here, the newly prepared DA complex DPTTA‐F_1_TCNQ together with the F_2_TCNQ and F_4_TCNQ based crystals were characterized with the fabrication of SCFETs for deriving the carrier mobilities. SCFETs with bottom‐gate top‐contact (BGTC) configuration were fabricated based on corresponding microcrystals growing on SiO_2_/Si substrates. The detailed description of the fabrication process is available in the reference.^6^ Here, the V‐shape FET transfer curves of all those devices demonstrate ambipolar characters of these crystals (**Figure** 2a–c). The mobility was calculated to be 0.15 cm^2^ V^−1^ s^−1^ (μ_h_), 0.24 cm^2^ V^−1^ s^−1^ (μ_e_) for DPTTA‐F_1_TCNQ; 1.01 cm^2^ V^−1^ s^−1^ (μ_h_), 0.27 cm^2^ V^−1^ s^−1^ (μ_e_) for DPTTA‐F_2_TCNQ; and 0.11 cm^2^ V^−1^ s^−1^ (μ_h_), 0.46 cm^2^ V^−1^ s^−1^ (μ_e_) for DPTTA‐F_4_TCNQ. Compared to the results reported previously, the value of μ_h_ and μ_e_ of DPTTA‐F_2_TCNQ and DPTTA‐F_4_TCNQ is slightly lower, but still in a reasonable range considering the quality variation of the solution processed single crystals. The P channel and N channel threshold voltage (*V*_th_) is −1.2 V, 18.4 V for DPTTA‐F_1_TCNQ; 43.7 V, −117.4 V for DPTTA‐F_2_TCNQ; 44.1 V, −43.1 V for DPTTA‐F_4_TCNQ respectively. The corresponding output curves of transistors based on DPTTA‐F_1_TCNQ, DPTTA‐F_2_TCNQ, and DPTTA‐F_4_TCNQ single crystals are presented in Figure S2 in the Supporting Information. Figure S3 in the Supporting Information shows the on/off current ratios (*I*_on_/*I*_off_). The mobility distribution for these DPTTA based devices can be seen in Figure S4a,c,e in the Supporting Information. The structures of the microcrystals used for preparing devices were examined using X‐ray diffraction (XRD) (Figure 2d–f). Sharp Bragg reflections can be indexed according to the crystallographic data for the bulk crystals. For DPTTA‐F_1_TCNQ microcrystals, the peak at 8.32° corresponds well with that of (001) planes. For DPTTA‐F_2_TCNQ, the strong peaks at 8.24°, 16.41°, and 24.68° are indexed as (001), (002), and (003) respectively. In the case of DPTTA‐F_4_TCNQ, the strong peaks at 8.11°, 16.15°, and 16.39° correspond to (001), (002), and (003). The data show that the crystals all grow along a preferring direction parallel to the DA alternative stacking direction. The corresponding selected area diffraction patterns (SAED) of the microcrystals are shown in Figure S4b,d,f in the Supporting Information.

**Figure 2:**
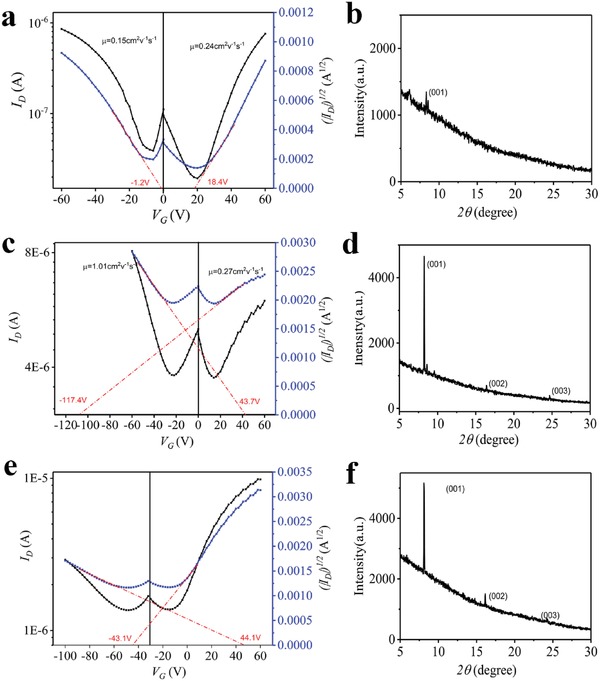
a–f) Transfer characteristics and the corresponding X‐ray diffraction: a,b) for DPTTA‐F_1_TCNQ, the ratio of channel width (*W*) and channel length (*L*), namely *W*/*L* = 0.38, |*V*_D_| = 50 V; c,d) for DPTTA‐F_2_TCNQ, *W*/*L* = 0.15, |*V*_D_| = 50 V; e,f) for DPTTA‐F_4_TCNQ, *W*/*L* = 0.27, |*V*_D_| = 50 V.

The conductivity can be defined as

(2) σ=nqμ

where σ is the conductivity, *n* is the charge carrier concentration, and μ is the carrier mobility.^20^ Here, the conductivity of those microcrystals is obtained with two‐probe method and we measured their length, width and thickness individually. They are calculated to be 0.0008, 0.07, 0.06 S cm^−1^ for DPTTA‐F_1_TCNQ, DPTTA‐F_2_TCNQ, and DPTTA‐F_4_TCNQ respectively. Before deriving the charge carrier concentration via applying Equation (2), a more detail analysis on the transfer and output curves of these devices should be carried out considering the low‐energy gap character of these ambipolar semiconductors. Electrons and holes generated through thermal activation will contribute to the conductivity simultaneously. The output curves in the range of −10 to 10 V were enlarged and shown in Figure S5 in the Supporting Information. It can be seen that the device of F_1_TCNQ complex displays the lowest off‐current in the range of 2 × 10^−10^–4 × 10^−10^ A (under a *V*_D_ of 10 V), corresponding to the largest energy gap of this material as the optical spectra revealed, which will be discussed below. Meanwhile the symmetric transfer curve of DPTTA‐F_1_TCNQ along *V*_G_ = 0, shows that ambipolar transport behavior is around zero gate voltage and the material possess relatively balanced hole and electron concentration.^21^ The F_2_TCNQ complex devices show similar situation, but a much higher off‐current in the range of 2 × 10^−7^–4 × 10^−7^ A with *V*_D_ = 10 V. The symmetric transfer curve and ambipolar transport character around *V*_G_ = 0 V show a balance bipolar state of this material. What observed with the transfer curves and output curves of F_4_TCNQ devices are quite different from that of previous two. The symmetric ambipolar transfer curve is only observed around the *V*_G_ of −30.6 V, showing that extra negative gate voltage is needed for inducing holes to compensate the dominating electrons. The electron domination behavior can be verified with the output characters in the p‐type regime, which shows depleting feature,^22^ the current decreases with the *V*_G_ applied from 0 to −60 V and get increasing again when *V*_G_ exceeding −80 V (Figure S5e, Supporting Information). This could be interpreted with the fact that when complexing with F_4_TCNQ, the strongest electron acceptor, the resulting material possess the deepest conducting band minimum (CBM) according to the band structure calculation as discussed below, which leads to the unintentional n‐doping by moisture from the ambient circumstance. So, when we apply Equation (2) for extracting the carrier concentration by combining the FET mobility and conductivity, it should be aware that both the electron and hole contributions should be addressed by applying the modified one

(3) $$\sigma =q(n_{e}\mu_{e}+n_{h}\mu_{h})$$

Here, *n*_e_/*n*_h_ and μ_e_/μ_h_ refer to the electron/hole concentration and mobilities, respectively.^23^ For DPTTA‐F_1_TCNQ and DPTTA‐F_2_TCNQ the balanced hole and electron density is calculated to be 1.31 × 10^16^ and 3.4 × 10^17^ cm^−3^, while a dominating electron density of 8.15 × 10^17^ cm^−3^ is estimated for DPTTA‐F_4_TCNQ (**Table** 1).

**Table 1:** The evolution of carrier density *n*, activation energy *E*_a_, bandgap *E*_g_, and energy difference between *E*_F_ and VBM and the work function with increasing F atoms in the DA complexes

Complexes	DPTTA‐F_1_TCNQ	DPTTA‐F_2_TCNQ	DPTTA‐F_4_TCNQ
*n* [cm^−3^]	1.02 × 10^16^ ^a)^	3.42 × 10^17^ ^b)^	8.15 × 10^17^ ^c)^
*E*_a_ [eV]	0.21	0.10	0.098
*E*_g_ [eV]	0.41	0.33	0.31
*E*_F_‐VBM [eV]	0.62	0.42	0.30
Work function [eV]	4.53	4.66	4.83

^a)^ Relatively balanced hole and electron for DPTTA‐F_1_TCNQ

^b)^ Balanced hole and electron for DPTTA‐F_2_TCNQ

^c)^ Electron for DPTTA‐F_4_TCNQ.

Seebeck coefficient measurement is a powerful tool for characterizing the semiconducting materials.^24^ The sign (positive or negative) of *S* directly reflects the polarity of the majority charge carrier (hole or electron) of unipolar materials. For bipolar materials, where electron and hole contribute to the electrical conductivity simultaneously, the overall Seebeck coefficient can be described by the conductivity weighted average according to the Equation (4)

(4) $$S=\frac{S_{h}\times \sigma_{h}+S_{e}\times \sigma_{e}}{\sigma_{h}+\sigma_{e}}$$

Here, *S*_h_ */S*_e_ and σ_h_ */σ*_e_ refer to the Seebeck and electrical conductivity of the two different carrier species.^25^ For nondegenerate materials with the Fermi level located at the middle of the bandgap, *n*_e_ = *n*_h_ and *S*_h_ = −*S*_e_.^26^ The sign of *S* is determined by the mobility ratio between electron and hole. It seems that balanced bipolar character supposed for DPTTA‐F_1_TCNQ is conflicting with the positive *S* measured (830 µV K^−1^), as the mobility of electron is slightly larger than that of hole according to the SCFET devices characterizations. This indicates that carrier concentration of hole and electron is deviating from equilibrium, with the hole concentration exceeding electron concentration in this crystal. As the mobilities of the two carrier species are close to each other, this nonequilibrium could not be observed through the FETs characterization. The measured seebeck coefficient is 185 µV K^−1^ for DPTTA‐F_2_TCNQ. The absolute value of *S*_e_ and *S*_h_ can be calculated to be 320 µV K^−1^ according to Equation (4). The overall Seebeck coefficient is significantly reduced comparing with that of the absolute *S* value of hole and electron in this ambipolar semiconductor, showing the detrimental bipolar effect on the TE performance. The negative Seebeck coefficient observed with DPTTA‐F_4_TCNQ is −443 µV K^−1^. The absolute value is obviously larger than that of DPTTA‐F_2_TCNQ showing the bipolar cancelling effect in this material has been minimized due to the dominant electron concentration induced by unintentional doping.^27^

To characterize the thermoelectric properties, the temperature dependence of the electrical conductivity and the Seebeck coefficient of those complexes were measured (**Figure** 3a,b). The samples for thermoelectric test were the same as those for single crystal structure analysis. Thermoelectric properties could be weighted using PF value. As depicted in Figure 3a–c. the electrical conductivities of the three cocrystals increased continuously with the testing temperature, while the absolute value of Seebeck coefficients continuously decreased with increasing temperature. The corresponding PFs displayed a positive dependence on temperature with the highest performance observed in DPTTA‐F_4_TCNQ. PF of DPTTA‐F_4_TCNQ is 0.33 µW m^−1^ K^−2^ at 300 K, which gradually increases to 0.62 µW m^−1^ K^−2^ at 400 K. The unintentional doping results in the electron dominant transport in F_4_TCNQ complex and suppress the bipolar effect on the Seebeck coefficient, which leads to highest TE performance in these DA complexes.

**Figure 3:**
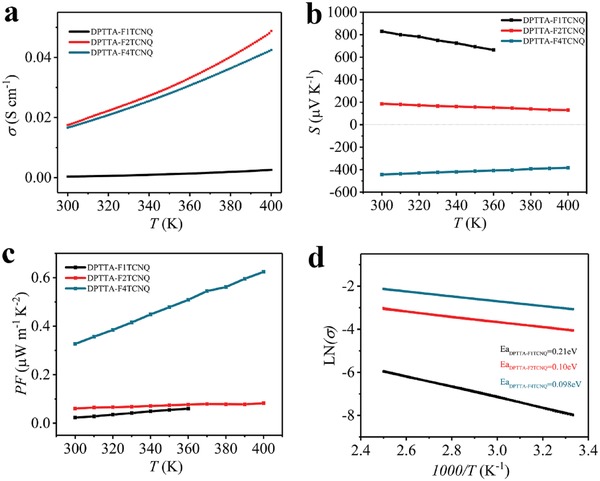
a) Temperature‐dependent conductivity and b) Seebeck coefficient for DPTTA‐F*_X_*TCNQ system. c) Temperature‐dependent (300–400 K) behavior of PF for the three complexes. d) LNσ as a function of *T* ^−1^ which revel *E*_a_.

The conductivity‐temperature dependence of all three complexes display typical thermal activated semiconducting behaviors. A linear relationship between lnσ and 1/*T* can be observed and fitted with the Arrhenius equation in the temperature range of 300–400 K (Figure 3d)

(5) $$\sigma \;=\sigma_{0}\;\exp\left(\frac{E_{a}}{KT}\right)$$

where σ is the conductivity, σ_0_ is a prefactor, *E*_a_ is the activation energy, *K* is Boltzmann constant, and *T* denotes absolute temperature.^23^ *E*_a_ reflects how easily charge carriers can be excited. The extracted activation energy of DPTTA‐F_1_TCNQ is 0.21 eV, which is about twice of DPTTA‐F_2_TCNQ (0.10 eV) and DPTTA‐F_4_TCNQ (0.098 eV) (Table 1). High activation energy leads to a low charge density and hence low electrical conductivity of DPTTA‐F_1_TCNQ.

The bandgap (*E*_g_) is a critical parameter determining the TE performance. Here the bandgap was determined using infrared spectroscopy (IR) by applying the Tauc method: (*αhν*)^*n*^ ∝ (*hν* − *E*_g_). In this equation, α is the absorption coefficient, *hν* is the photon energy, and *n* = 2 and 1/2 for direct and indirect gaps. *E*_g_ is derived from a linear extrapolation to zero absorption in the plot.^28^ As shown in **Figure** 4, the direct bandgap of the DPTTA‐F_1_TCNQ, DPTTA‐F_2_TCNQ, and DPTTA‐F_4_TCNQ are 0.41, 0.33, 0.31 eV respectively (Table 1). The bandgap displayed a decreasing tendency from DPTTA‐F_1_TCNQ to DPTTA‐F_4_TCNQ. This narrow bandgap feature is important for achieving high PF.

**Figure 4:**
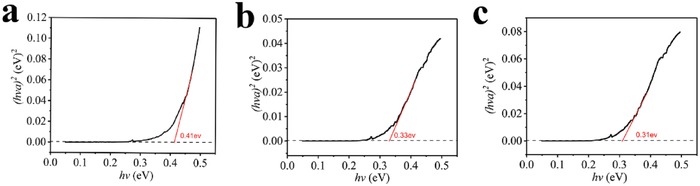
a–c) Optical spectroscopy characterization of Tauc plots of (*hνa*)^2^ versus photon energy (*hν*) for: a) DPTTA‐F_1_TCNQ, b) DPTTA‐F_2_TCNQ, and c) DPTTA‐F_4_TCNQ. A linear fit (red line) was used to estimate the bandgap by extrapolating to zero absorption.

**Figure** 5a shows the ultraviolet photoemission spectroscopy (UPS) presenting the evolution of work function and valance band edge in DPTTA‐F*_X_*TCNQ system. The shift in the cutoff indicates the differences in the work function of the cocrystals, which goes from 4.53 eV for DPTTA‐F_1_TCNQ to 4.83 eV for DPTTA‐F_4_TCNQ (Table 1). The valance band peak edge is as depicted in the right plots in Figure 5a, which illustrates that the valence band maximum (VBM) of DPTTA‐F_1_TCNQ, DPTTA‐F_2_TCNQ, and DPTTA‐F_4_TCNQ are located at 0.62, 0.42, and 0.30 eV (Table 1) below *E*_F_ respectively. These data show a tendency that the VBM of the resulting complexes decrease with the increase of electron affinity of the acceptor component.

**Figure 5:**
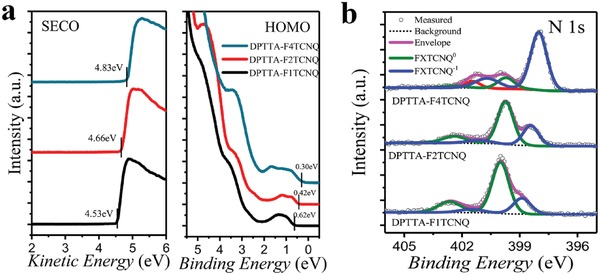
a) Evolution of work function and valence band edge with the increasing number of F atoms in the DPTTA‐F*_X_*TCNQ system and b) XPS focusing on the N 1s region for DPTTA‐F*_X_*TCNQ complexes.

Figure 5b shows the X‐ray photoelectron spectroscopy spectra (XPS) of N1s for the three resulting complexes. The N1s spectra of the complexes indicate that there are two different chemical environments of N atoms existing in the samples, from which we can identify that the three complexes are composed of pristine acceptors, that is F_1_TCNQ^0^, F_2_TCNQ^0^, F_4_TCNQ^0^, and the corresponding anions, that is F_1_TCNQ^−1^, F_2_TCNQ^−1^, F_4_TCNQ^−1^, respectively. The ratio of F*_X_*TCNQ^−1^ and F*_X_*TCNQ^0^ from F_1_TCNQ to F_4_TCNQ is gradually increasing, indicating that the CT degree increases by adding more F atoms in acceptor molecules, which is consistent with the result discussed in the single crystal structure characterization.

To further understand the electrical transport properties of these CT complex crystals, quantum simulation of the superexchange effect including intermolecular electronic coupling as well as the band structure calculation were carried out. Formation of DA complexes has been wide accepted for explaining the low doping efficiency of organic semiconductors.^26^, ^29^ Here, the DA complex single crystal is the extreme situation of this doping process. When we investigate the isolated DA couples, hybridization of frontier orbitals and energy splitting of the resulting complexes could be observed. The energy difference of HOMO and LUMO level of the hybridized system is around 1.0 eV (**Table** **2**), obviously larger than that of the offset between the HOMO of donor and LUMO of acceptors.^6^, ^30^ Low charge carrier generation efficiency can be expected when this happens in the matrix of the corresponding organic semiconductors. For the 3D crystals DA complex, the electronic couplings between the hybrid frontier orbitals will lead to highly dispersed valance and conducting bands, as well as a narrow bandgap as illustrated in **Figure** 6.^10^, ^31^ It has already been well established that the superexchange effect plays a critical role in the mixed‐stack donor–acceptor complex crystals, which is determined by the strength of intermolecular electronic coupling and energy alignment of the frontier orbitals between the donor and acceptor components.^5^ Here, the employment of a series of TCNQ derivatives give the opportunity of tuning the electronic structure and hence the transport properties of the resulting complexes systematically. The effective electronic couplings between the donor (acceptor) molecules along the stacking direction through the superexchange interaction bridged by the intermediate acceptor (donor) molecules are calculated by building a DAD (ADA) cluster with an energy splitting method.^4^, ^10^ The results show a gradual increase of the transfer integral for holes and electrons (Table 2) with the increase of the F atoms presenting in the acceptor molecules, and which is consistent with the extension of the dispersive valance and conducting band calculated based on the single crystal structure (Figure S6, Supporting Information). All these results imply the efficient transport properties of these DA complexes. The roughly increasing tendency of carrier mobilities observed in SCFETs is coincident with the theoretical simulations, as the electronic couplings along the stacking direction plays a dominant role in the charge transport of these materials which is also the preferring growth direction of the single crystals used for FET fabrications. The absolute values of Fermi level (from −1.30, −1.56 to −1.66 eV for DPTTA‐F_1_TCNQ, DPTTA‐F_2_TCNQ to DPTTA‐F_4_TCNQ) go deeper according the calculation (Table 2), which is verified by the UPS characterization, as the vacuum levels continuously shift to higher kinetic energy showing the increase of work functions with more F atoms presenting in the TCNQ skeleton. This result shows the influence of electron deficient character of the F substitutions on the DA complexes, which will determine the majority carrier species of the materials. For F_1_TCNQ complex with the highest VBM level, it is more likely to be p‐type doped. For F_4_TCNQ complex with the deepest CBM level, it will be easily n‐type doped.

**Table 2:** The calculated energy level of frontier molecular orbitals (MO) of the isolated DPTTA‐F*_X_*TCNQ complexes; effective electronic coupling along the stacking direction; VBM and CBM and bandgap (*E*_g_ = CBM‐VBM). Energy in meV

	MO of the complexes		*t*_hole_	*t*_electron_	VBM	CBM	*E*_g_
	*H*_DA_	*L*_DA_					
DPTTA‐F_1_TCNQ	−4932	−4059	76.7	71.6	−1297	−1205	92
DPTTA‐F_2_TCNQ	−4987	−3978	80.3	89.0	−1563	−1486	77
DPTTA‐F_4_TCNQ	−5056	−4065	84.4	95.4	−1666	−1631	35

**Figure 6:**
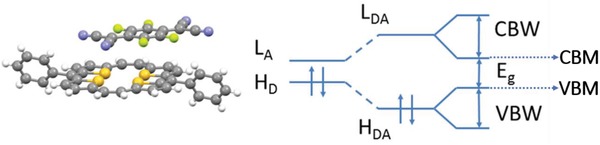
Schematic drawing of the band structure of DPTTA‐F*_X_*TCNQ complexes. *L*_A_ and *H*_D_ refer to LUMO of isolated acceptor and HOMO of donor molecules, respectively. *L*_DA_ and *H*_DA_ refer to LUMO and HOMO of the DA complex. CBW and VBW refer to conducting band width and valance band width. CBM and VBM refer to conducting band minimum and valance band maximum.

This is consistent with the observed switching of the sign of Seebeck coefficients in these materials. The narrow bandgap and the decreasing tendency from F_1_TCNQ (92 meV) to F_4_TCNQ (35 meV) complexes were also reproduced in the quantum simulation (Table 2). The deviation in the absolute value compared to the experimental data is due to the fact that the bandgap is usually underestimated with the DFT method.

Isostructural DA complexes formed by DPTTA and F*_X_*TCNQ (*X* = 1, 2, 4) display narrow optical bandgap in the range of 0.31 to 0.41 eV, and continuous down shift of the VBM and CBM. This leads to a thermal activated bipolar character and majority carrier switching from hole dominated semiconductor in F_1_TCNQ complex to electron dominated semiconductor in F_4_TCNQ complex. This result shows that tuning of the molecular frontier energy level and the intermolecular interactions including the charge transfer degree, intermolecular electronic coupling can be easily realized in the DA complex systems, which leads to the efficient tuning of the band structure of organic semiconductors. Unintentional doping leads to the break of balanced bipolar transport which is important for achieving a high TE performance in these narrow bandgap organic semiconductors, similar to that of the inorganic counterpart.

## Experimental Section

*Material and Crystal*: DPTTA was purchased from Solarmer Beijing. F_1_TCNQ, F_2_TCNQ, and F_4_TCNQ were purchased from TCI. They were all used without further purification.

Single crystals based on DPTTA suitable for X‐ray single crystal structure characterization and measurement of thermoelectric properties were obtained through diffusion method. A solution of DPTTA was mixed in chlorobenzene and the corresponding acceptor (molar ratio 1:1) in the same solvent. After 24 h, the black shiny crystals were collected which can be used for IR, XPS, and UPS measurement. Redissolving the obtained crystals in chlorobenzene to get their saturated solutions. Then microcrystals suitable for FET device fabrication could be obtained by drop casting of the saturated solution onto the SiO_2_/Si substrates.

*Structure Characterization and Measurements*: IR spectra were investigated using a TENSOR‐27 spectrometer (Bruker). XPS and UPS were performed by using an AXIS Ultra‐DLD ultrahigh vacuum photoemission spectroscopy system (Kratos Co.). TEM and SAED measurements were carried out on a JEM 2011 (JEOL). XRD was measured on Empyrean (PANalytical B.V.) with CuKα source (λ = 1.541 Å).X‐ray crystallographic data were collected on ST Saturn724+ (Rigaku), using CuKα radiation (λ = 1.541 Å).

*I–V* characteristics of the SCFETs were recorded with a Keithley 4200 SCS and a Micromanipulator 6150 probe station at room temperature in air. The mobilities were calculated using Equation (6) as follows

(6) $$I_{D}=\mu C_{i}\left(W/2L\right){\left(V_{G}-V_{\mathrm{th}}\right)}^{2}$$

where *I*_D_ is the drain current, *C*_i_ is the capacitance per unit area of insulating layer which was 11 nF cm^−1^ in the experiment, *L* is the channel length, *W* is the channel width and *V*_th_ is the threshold voltage.

The measurement of thermoelectric parameters (electrical conductivity and Seebeck coefficient) of the crystals was all carried out in vacuum. Electrical conductivity was measured by a standard four‐probe method using a Physical Property Measurement Systems instrument (PPMS DynaCool 9T, Quantum design). Striped patterns (300 µm interval) of gold electrodes (with thicknesses of 60 nm) were thermally evaporated onto the upper surface of the selected crystals (about 0.3 × 3 mm) then they were mounted on measuring boards via the connection of gold wires with a diameter of 25 µm and fixed with gold conductive paint (EMS). Seebeck coefficient was measured using the SB‐100 Seebeck Measurement System (MMR Tech.) for DPTTA‐F_2_TCNQ and DPTTA‐F_4_TCNQ. The measurement of DPTTA‐F_1_TCNQ was unreliable in this system due to voltage overload, so it was measured with SB‐C‐01 Seebeck Measurement System (Institute of Electrics, Chinese Academy of Sciences). Sample crystals (about 0.3 × 3 mm) were fixed on the measuring boards with conductive carbon paint (SPI).

*Quantum Simulation*: Electronic structure calculations on the isolated DA complexes, ADA and DAD triads have been performed through B3LYP functional and 6–31G(d) basis sets implemented in Gaussian09. Band structure calculation was carried out by using the plane‐wave technique as implemented in CASTEP code. An 800 eV cutoff energy was used for the plane‐wave basis set, and norm conserving pseudopotentials were employed for all ions. The exchange correlation energy is described by the generalized gradient approximation (GGA) in the form of Perdew, Burke, and Ernzerhof (PBE).

## Conflict of Interest

The authors declare no conflict of interest.

## Supporting information

Supporting Information
