# Supplementary material for: Band Engineering and Majority Carrier Switching in Isostructural Donor–Acceptor Complexes DPTTA‐FXTCNQ Crystals (X = 1, 2, 4)
Source: Adv Sci (Weinh). 2019 Nov 26;7(3):1902456. doi: 10.1002/advs.201902456 (PMC7001638; doi:10.1002/advs.201902456)
Supplement: Supplementary file 1 — Supporting Information [file ADVS-7-1902456-s001.pdf]

Copyright WILEY-VCH Verlag GmbH & Co. KGaA, 69469 Weinheim, Germany, 2019.

Supporting Information

for *Adv. Sci.,* DOI: 10.1002/advs.201902456

Band Engineering and Majority Carrier Switching in Isostructural Donor–Acceptor Complexes DPTTA-F_XTCNQ Crystals (X = 1, 2, 4)

Yingying Liang, Yunke Qin, Jie Chen, Weilong Xing, Ye Zou, Yimeng Sun, Wei Xu, and Daoben Zhu*

Copyright WILEY-VCH Verlag GmbH & Co. KGaA, 69469 Weinheim, Germany, 2019.

Supporting Information

Band Engineering and Majority Carrier Switching in Isostructural Donor-Acceptor Complexes DPTTA-F_XTCNQ Crystals (X = 1, 2, 4)

Yingying Liang, Yunke Qin, Jie Chen, Weilong Xing, Ye Zou, Yimeng Sun, Wei Xu, Daoben Zhu*

Table S1. Crystallographic data for DPTTA based DA complexes

	DPTTA-F1TCNQ	DPTTA-F2TCNQ	DPTTA-F4TCNQ
Empirical formula	C46 H24 F N4 S4	C46 H24 F2 N4 S4	C46 H22 F4 N4 S4
Formula weight	779.93	798.93	834.91
Temperature	293(2) K	293(2) K	173.1500 K
Wavelength	0.71073 Å	0.71073 Å	0.71073 Å
Crystal system	Triclinic	Triclinic	Triclinic
Space group	$P\bar{1}$	$P\bar{1}$	$P\bar{1}$
Unit cell dimensions	a = 7.7633(16) Å; α=73.11(3)°	a = 7.9501(8)Å; α= 88.974(11)°	a=8.0037(17)Å;α=88.144(7)°
	b = 10.972(2) Å; β= 80.23(3)°	b=10.4022(14)Å;β=80.486(10)°	b=10.130(2)Å; β=81.179(6)°.
	c = 11.388(2) Å; γ=79.92(3)°	c=11.0500(15)Å; γ=82.039(10)°	c = 11.250(2) Å; γ=82.289(6)°.
Volume	906.5(4) Å^3	892.6(2) Å^3	893.1(3) Å^3
Z	1	1	1
Density (calculated)	1.429 Mg/m^3	1.486 Mg/m^3	1.552 Mg/m^3
Absorption coefficient	0.309 mm^{-1}	0.320 mm^{-1}	0.331 mm^{-1}
F(000)	401	410	426
Theta range for data collection	3.061 to 27.491°	2.623 to 27.499°	2.029 to 27.468°
Index ranges	-10<=h<=9, -14<=k<=14, -14<=l<=14	-10<=h<=9, -13<=k<=13, -14<=l<=14	-10<=h<=10, -13<=k<=13, -14<=l<=14
Reflections collected	12566	11839	12294
Independent reflections	4122 [R(int) = 0.0558]	4084 [R(int) = 0.0754]	4063 [R(int) = 0.0351]
Completeness to theta = 25.242°	99.8 %	99.9 %	99.4 %
Absorption correction	None	None	Semi-empirical from equivalents
Refinement method	Full-matrix least-squares on F^2	Full-matrix least-squares on F^2	Full-matrix least-squares on F^2
Data/restraints / parameters	4122 / 0 / 253	4084 / 0 / 253	4063 / 0 / 262
Goodness-of-fit on F^2	1.170	1.065	1.083

Final R indices [I>2sigma(I)]	R1 = 0.0617, wR2 = 0.1463	R1 = 0.0759, wR2 = 0.1700	R1 = 0.0384, wR2 = 0.0900
R indices (all data)	R1 = 0.0664, wR2 = 0.1497	R1 = 0.1186, wR2 = 0.1985	R1 = 0.0425, wR2 = 0.0925
Extinction coefficient	n/a	n/a	n/a
Largest diff. peak and hole	1.540 and -0.287 e.$Å^{-3}$	1.050 and -0.485 e.$Å^{-3}$	0.329 and -0.217 e.$Å^{-3}$

Table S2. CT characteristics of the complexes evaluated from changes in the bond lengths.

	r_0[a]	r_{-1}[a]	r_{CT}	CT
DPTTA-F_1TCNQ	0.4785[b]	0.4974[c]	0.4806	0.111
DPTTA-F_2TCNQ	0.4778[d]	0.4956[e]	0.4816	0.213
DPTTA-F_4TCNQ	0.4779[f]	0.5[g]	0.4983	0.923

a r_0 and r_{-1} are the values for the neutral acceptor and for the fully ionized acceptor, respectively.

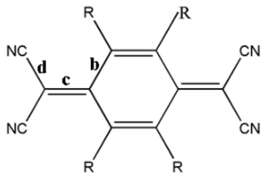

$r = c/(b + d)$

b, d[1]; c [2]; e f [3]; g[4].

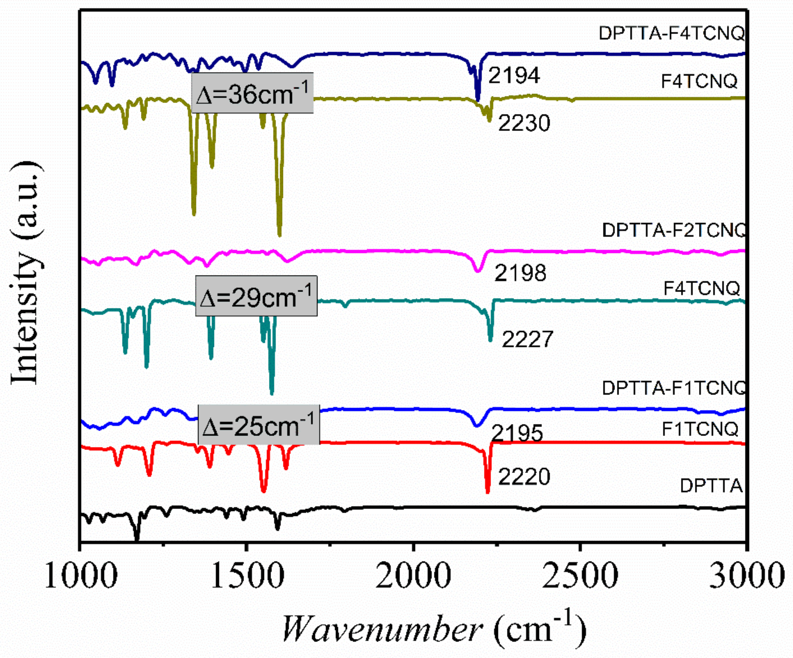

Figure S1. IR spectra of DPTTA-F_1TCNQ, DPTTA-F_2TCNQ, and DPTTA-F_4TCNQ compared with pristine DPTTA and F_XTCNQ at room temperature. The shift of C≡N stretching vibration from 25cm^{-1} of F_1TCNQ to 29 cm^{-1} and 36cm^{-1} for F_2TCNQ and F_4TCNQ indicates the increase of charge transfer degree between the corresponding donor and acceptor molecules.

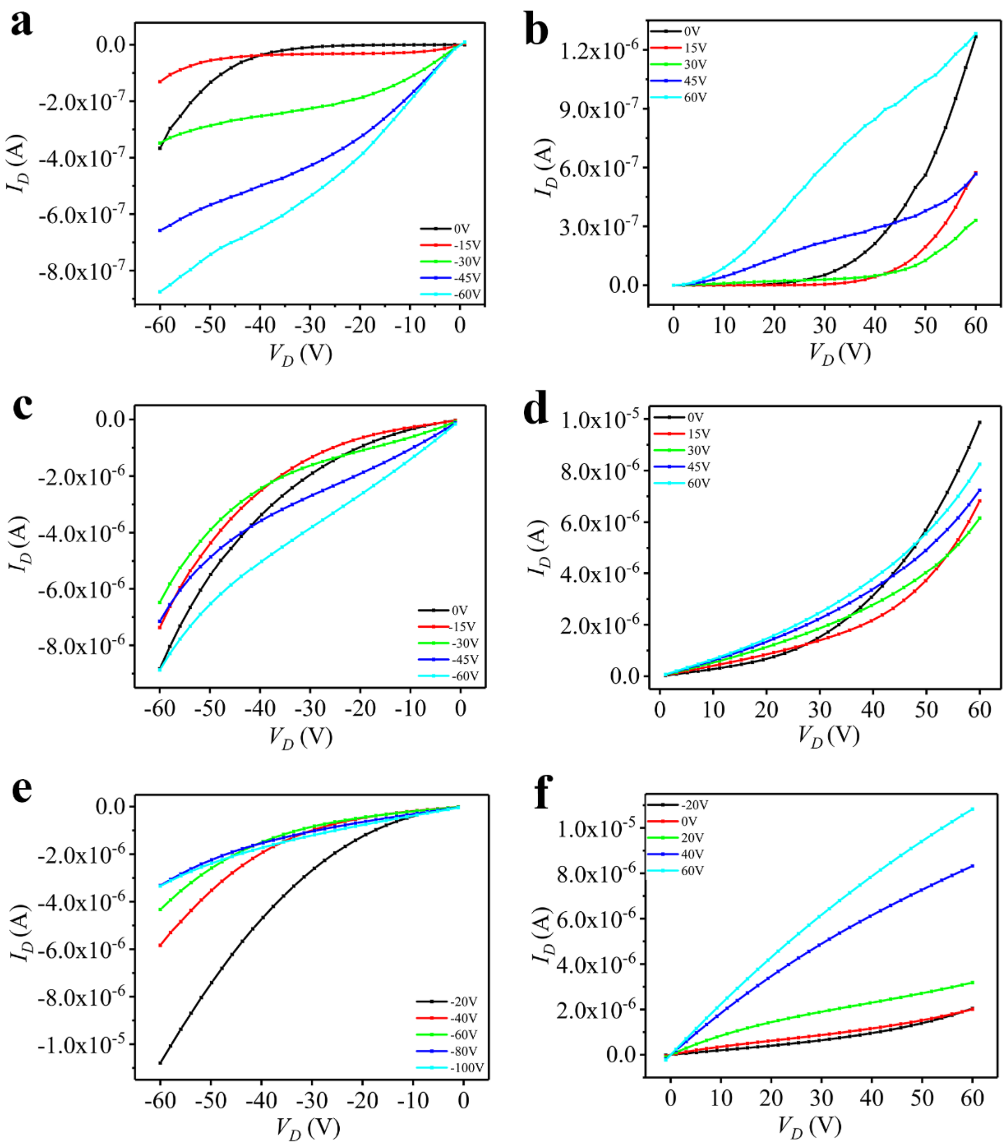

Figure S2. Output curves of DPTTA-based complexes, corresponding to the transfer curves presented in the text. (a, b) for DPTTA-F_1TCNQ; (c, d) for DPTTA-F_2TCNQ; (e, f) for DPTTA-F_4TCNQ.

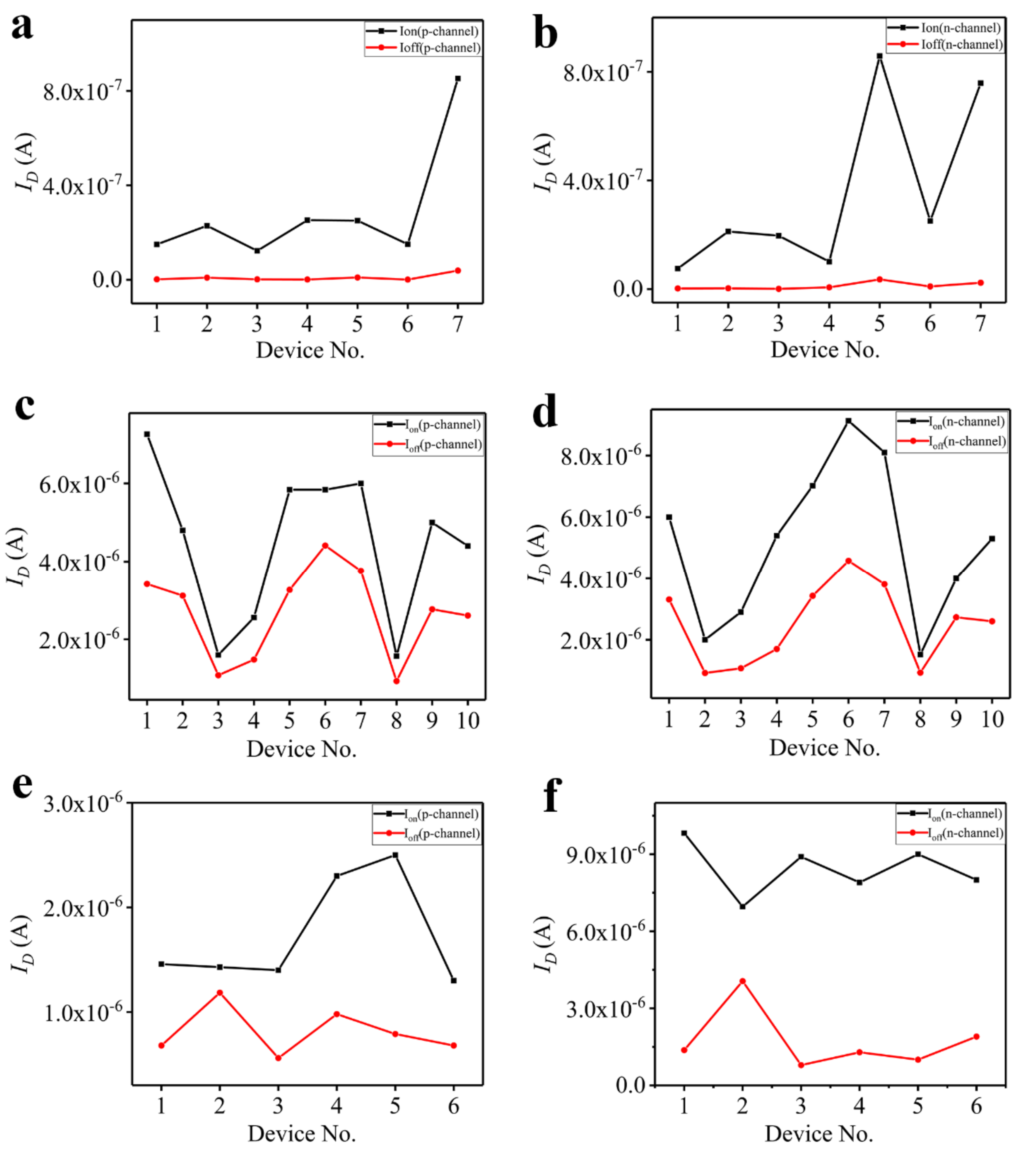

Figure S3. The I_{on} and I_{off} distribution of DPTTA-based complex devices. The on/off current ratios (I_{on}/I_{off}) are all less than 10^2 orders of magnitude, even lower than 10 for DPTTA- F_2TCNQ and DPTTA-F_4TCNQ devices. (a, b) for DPTTA-F_1TCNQ; (c, d) for DPTTA-F_2TCNQ; (e, f) for DPTTA-F_4TCNQ.

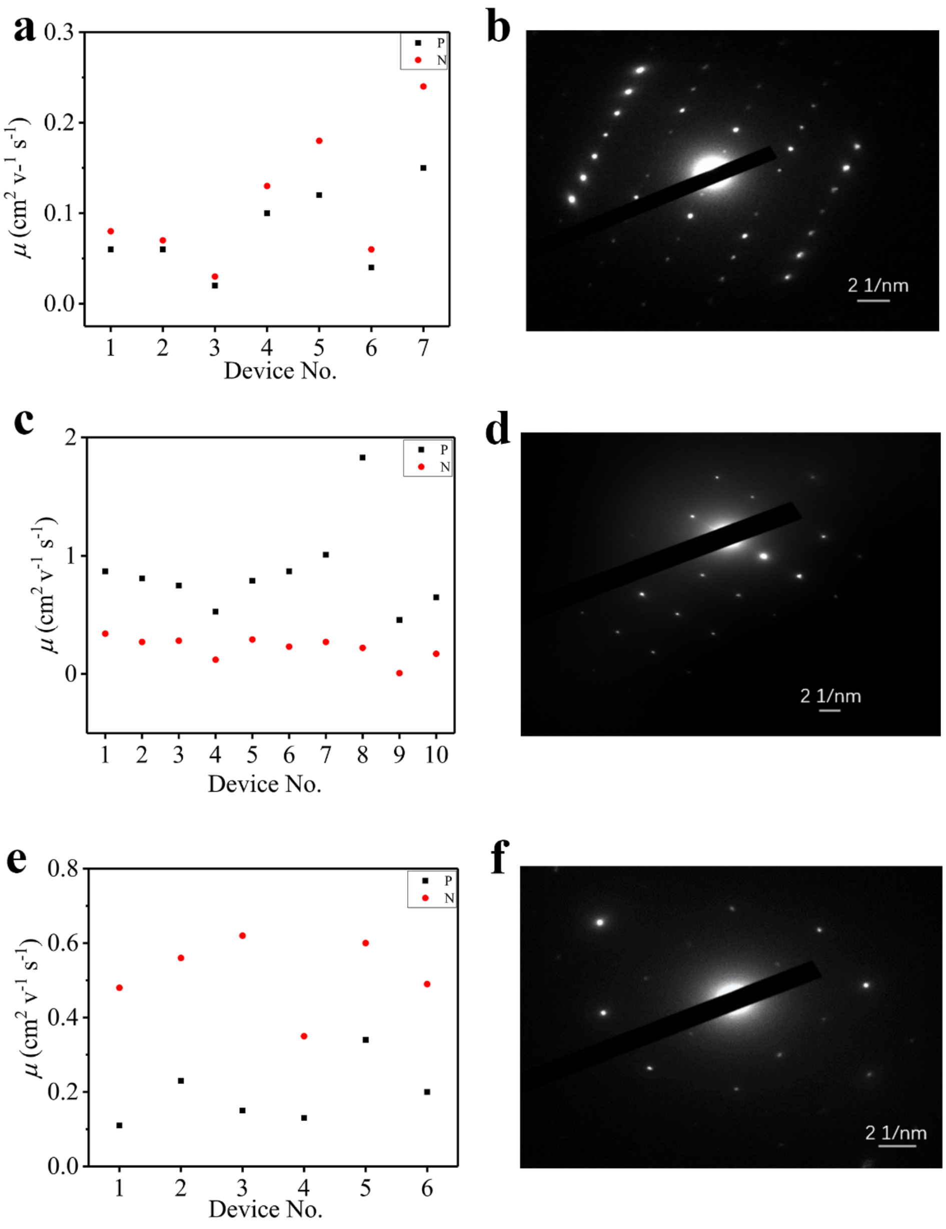

Figure S4. Mobility distribution of DPTTA-based complex devices and corresponding diffraction patterns of microcrystals. (a, b) for DPTTA-F_1TCNQ; (c, d) for DPTTA-F_2TCNQ; (e, f) for DPTTA-F_4TCNQ.

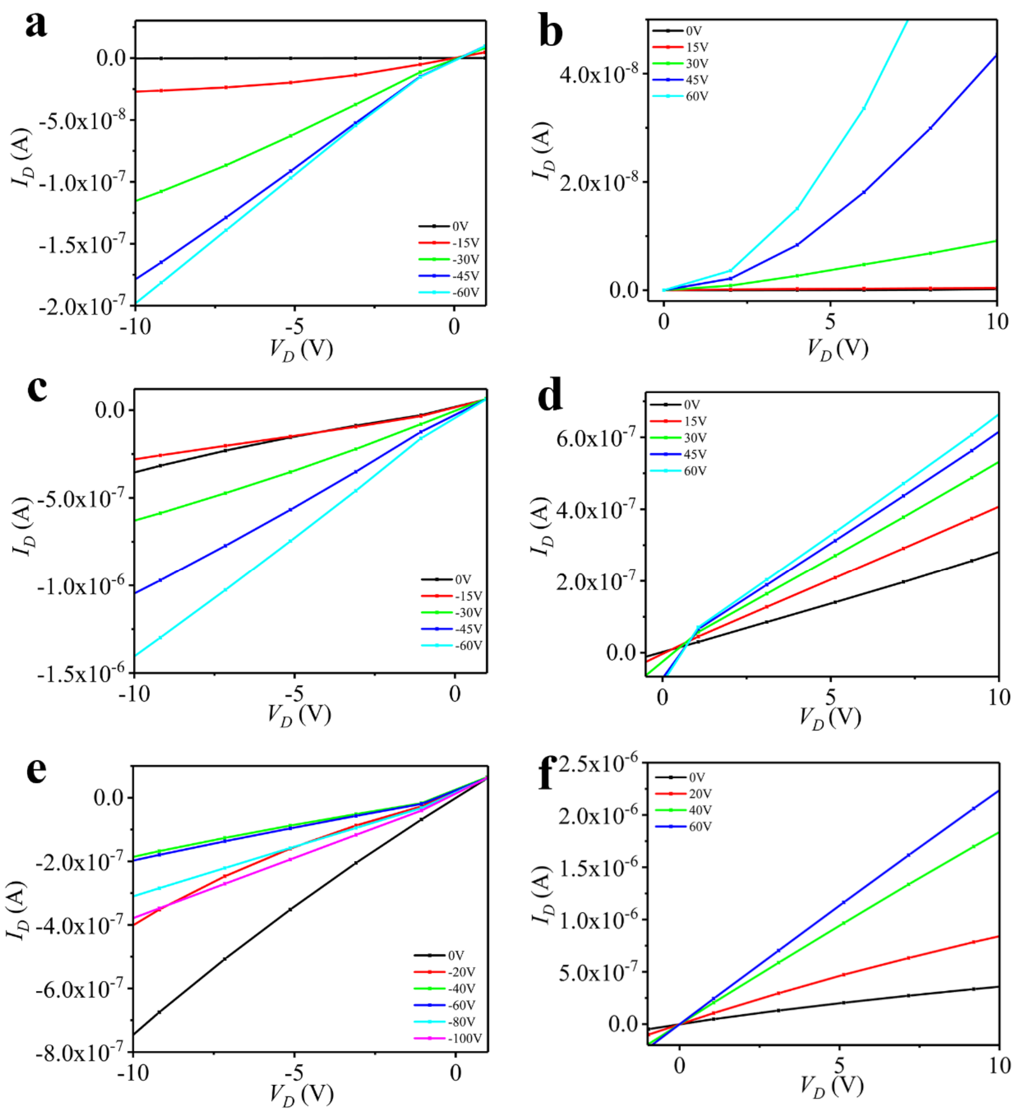
a
b
c
d
e
f
I_D (A)
V_D (V)
0V
-15V
-30V
-45V
-60V
15V
30V
45V
60V
-20V
-40V
-60V
-80V
-100V
20V
40V
60V

Figure S5. Enlarged output curves of FET devices of DPTTA-F_xTCNQs with the V_D in the range of -10 to 10 V. (a, b) for DPTTA-F_1TCNQ; (c, d) for DPTTA-F_2TCNQ; (e, f) for DPTTA-F_4TCNQ.

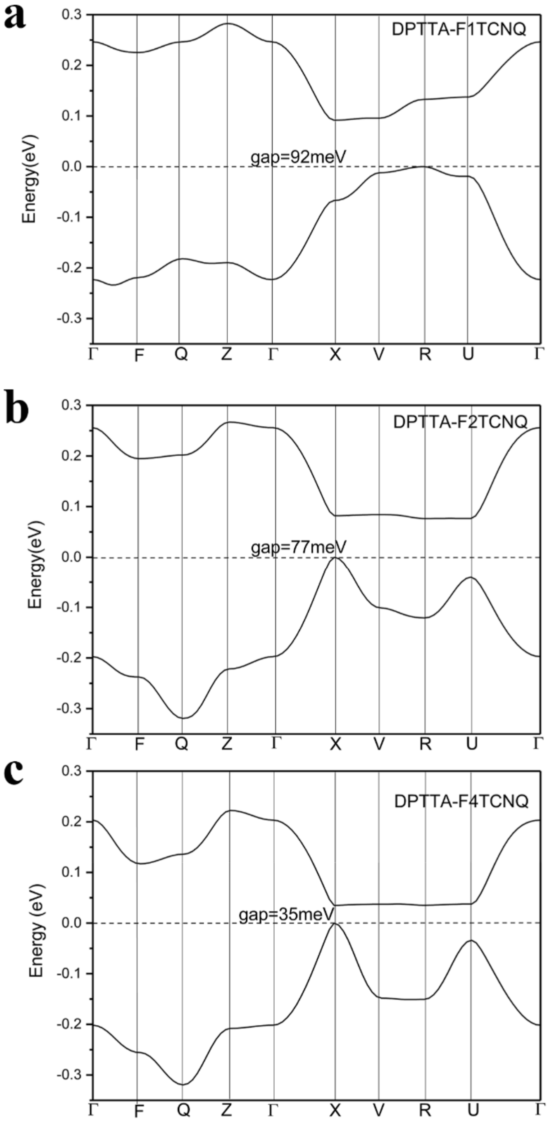

Figure S6. Band structures and gap (Eg = CBM-VBM) of the DPTTA-FxTCNQ (x=1, 2, 4). The reciprocal coordinates of high-symmetry points are Γ=(0, 0, 0), F=(0, 0.5, 0), Q=(0, 0.5, 0.5), Z =(0, 0, 0.5), X=(0.5, 0, 0), V=(0.5, 0.5, 0), R=(0.5, 0.5, 0.5), U=(0.5, 0, 0.5).

Reference

[1] F. M. Wiygul, T. J. Emge, J. P. Ferraris, T. J. Kistenmacher, *Mol. Cryst. Liq. Cryst.* **1981**, *71*, 303.

[2] T. Murata, G. Saito, K. Nakamura, M. Maesato, T. Hiramatsu, Y. Yoshida, *Cryst. Growth Des.* **2013**, *13*, 2778.

[3] T. J. Emge, M. Maxfield, D. O. Cowan, T. J. Kistenmacher, *Mol. Cryst. Liq. Cryst.* **1981**, *65*, 161.

[4] J. F. F. Jose-Larong, Y. Takahashi, T. Inabe, *Struct. Chem.* **2013**, *24*, 113.
